# Reading Skills of Children with Dyslexia Improved Less Than Expected during the COVID-19 Lockdown in Italy

**DOI:** 10.3390/children8070560

**Published:** 2021-06-29

**Authors:** Ilaria Maria Carlotta Baschenis, Laura Farinotti, Elena Zavani, Serena Grumi, Patrizia Bernasconi, Enrica Rosso, Livio Provenzi, Renato Borgatti, Cristiano Termine, Matteo Chiappedi

**Affiliations:** 1 Child Neuropsychiatry Unit, IRCCS Mondino Foundation, 27100 Pavia, Italy; ilaria.baschenis@mondino.it (I.M.C.B.); laura.farinotti@mondino.it (L.F.); serena.grumi@mondino.it (S.G.); patrizia.bernasconi@mondino.it (P.B.); enrica.rosso@mondino.it (E.R.); livio.provenzi@mondino.it (L.P.); renato.borgatti@mondino.it (R.B.); 2 Department of Brain and Behavioral Sciences, University of Pavia, 27100 Pavia, Italy; elena.zavani01@universitadipavia.it; 3 Department of Medicine and Surgery, University of Insubria, 21100 Varese, Italy; cristiano.termine@uninsubria.it

**Keywords:** COVID-19, dyslexia, reading skills, learning disability

## Abstract

Following school closures due to the SARS-CoV-2 pandemic, for some months, children received only distance learning. The effects of this approach, however, are not clear for children with dyslexia. We conducted a cross-sectional comparison between children with and without dyslexia after the so-called “lockdown” and a comparison between pre- and post-lockdown parameters in children with dyslexia. We recruited sixty-five children with dyslexia (dyslexia group, DG) from an outpatient facility in Pavia (Lombardy, Italy) and fifty-two children without specific learning disabilities as the control group (CG) from summer camps in the same province. We performed neuropsychological tests to explore reading skills and an ad hoc questionnaire to explore how parents and children had experienced the measures taken to reduce spreading of SARS-CoV-2 infection. Between 59 to 63% of children with dyslexia did not reach the average expected increase of reading skills. According to their parents, they also showed greater social isolation and fewer worries about the pandemic and the school’s closure. Our data indicate that children with dyslexia are at increased risk of consequences on their learning potential in case of school closure. They also seem to have a peculiar psychological experience of school closure. Specific interventions should therefore be provided to minimize the risk of negative effects on global development.

## 1. Introduction

During the first months of 2020, Italy was rapidly and dramatically affected by the rapid outbreak of the SARS-CoV-2 pandemic [1]. Government attempts to mitigate and contain the virus spread required a massive reduction of physical contact and the lockdown that followed also included the suspension of school activities from February 24th to the end of the academic year in June 2020. Distance learning was recommended [2], but its implementation was not immediate for many institutes and the delivery modes varied during the lockdown months and among different schools [3].

In this scenario, a reduction of learning outcomes may be expected as reported in previous studies on partial or temporary interruptions of school attendance [4]. Nonetheless, more negative consequences may be expected for children who already presented special educational needs and learning disabilities [3]. According to the latest edition of the Diagnostic and Statistic Manual of Mental Disorders (DSM-5 [5]), specific learning disorder is a neurodevelopmental disorder with biological origin, characterized by persistent difficulties in learning and using academic skills. This diagnosis does not apply to subjects with intellectual disabilities, uncorrected visual or auditory ability, other mental or neurological disorders, significant psychosocial adversities, inadequate language skills, or inadequate educational instruction. Dyslexia refers specifically to an impairment in word recognition, decoding, and spelling. Children with dyslexia require specific supports to reach adequate and satisfying learning goals. Besides rehabilitative attention [6], they need to receive specific teaching and to use compensative strategies and tools according to the specific needs of the single individual [7].

As such, as their educational needs require continuous dedicated and systematic care on a daily basis, children diagnosed with dyslexia may be at increased risk for detrimental learning consequences during the COVID-19 lockdown. The suspension of school in-presence activities may had impacted the continuity of educational care for children with dyslexia with the risk of increased emotional and psychological burden related to social distances, lack of dedicated specialist support, and isolation [3]. Moreover, during the lockdown, the engagement of parents in their children school activities dramatically increased as—in many case—they had to shift from working hours to a 24/7 care for their children’s special educational needs at home [8]. Parents of children with special educational needs exhibited worries about the possibility for their children to fall even further behind in school because they did not feel adequate to meet their specific needs during the COVID-19 emergency [9]. Additionally, worries about the lack of supervised and specialist care for their children’s disability condition and rehabilitation was the most significant predictor of parents’ stress, depressive and anxious symptoms during the COVID-19 lockdown [10]. 

Although they should be considered as a specific vulnerable population during the COVID-19 emergency, to the best of our knowledge, no study to date has reported on the effects of school suspension and distance learning in children with dyslexia. In the present study, we compared the reading skills (i.e., accuracy and speed) of a sample of children with dyslexia before (T1) and after the COVID-19 lockdown (T2) with a control group at T2. A preliminary analysis was conducted to control for differences in the perception and experience of the lockdown and distance learning in the two groups. Then, we analyzed the presence of significant changes in reading skills between T1 and T2 within the group of children with dyslexia. In order to better explore the clinical relevance of significant changes in reading skills within dyslexic children, they were compared with two different parameters: the reading skills of typically developing counterparts and the expected one-year improvement in reading speed.

## 2. Materials and Methods

### 2.1. Participants

Sixty-five children (*n* = 20 females) with dyslexia (dyslexia group, DG) were enrolled consecutively at the Child Neuropsychiatry Unit of the IRCCS Mondino Foundation, Pavia, Italy. Children were included if they were students from the third to the eighth grade, if they had had a previous reading assessment in 2019, if they were monolingual, and in the absence of neurological and psychiatric comorbidities. Fifty-two (*n* = 20 females) children without specific learning disabilities as the control group (CG) were enrolled at a summer camp in Pavia from July to August 2020. Neither group physically attended the school from March to June 2020 due to the COVID-19 lockdown.

The mean age of children of the dyslexia group (DG) was 10.64 years (Standard Deviation (SD) = 1.60, range = 8–14), while that of the control group (CG) was 9.80 years (SD = 1.57, range = 7–13). The *t*-test showed that CG children were significantly younger compared to the DG counterparts (*t* = 3.14, *p* < 0.01), therefore in the subsequent comparison analyses on reading abilities, the age was controlled as the covariate.

### 2.2. Procedures

Both groups completed three tasks to assess their reading skills (see below) and they filled in a questionnaire about online school characteristics and challenges. DG children were evaluated for reading skills two times: before the lockdown in 2019 (T1) and after the lockdown (T2). CG children were evaluated only once at T2. In consideration of the reading difficulties of DG subjects, the questionnaire was administered by a nominated researcher to the children of both groups. Additionally, parents filled in a self-report questionnaire on online school management.

### 2.3. Measures

Reading assessment. The reading assessment was performed through three tasks. The first two tasks (i.e., reading aloud a list of words and a list of non-words) were derived from the Battery for the Assessment of Developmental Dyslexia and Dysorthography-2 (DDE-2 Battery by Sartori et al. [11]) to assess reading speed (syllables per second) and accuracy (number of errors). The third task was derived from the Assessment of Reading and Comprehension Skills for Elementary and Middle School (MT-3-Clinic tasks by Cornoldi and Carretti [12]) and it consists of reading a text aloud to assess reading speed (syllables per second) and accuracy (number of errors).

Ad hoc questionnaire. An ad hoc questionnaire provided a detailed characterization of the online school delivered during the lockdown period (March–June 2020), including which kinds of remote education were implemented (e.g., online vs. pre-recorded lessons), major challenges in managing online school (e.g., online platform, connection, family management), and parents’ perception of their children learning trajectories. The questionnaire was filled in by parents as well as by children with the help of a dedicated researcher.

### 2.4. Plan of Analysis

Preliminary descriptive statistics were performed and the two groups of students were compared for demographic characteristics through an independent-sample *t*-test. Then, a set of *χ*^2^ tests were performed in order to identify differences in the survey’s results between the DG and CG subjects. In order to assess reading skills development of DG children, three different sets of analyses were used. First, T1-to-T2 differences in reading speed and accuracy (for words, non-words, and text) were tested in DG children through paired-sample t-tests. Second, two sets of analysis of covariance (ANCOVA) with age as the covariate were implemented to compare reading speed and accuracy between DG and CG children at T2. Third, a sub-group analysis was used to identify percentages of DG children that reached the expected one-year improvement in reading speed in Italian untreated dyslexic students, i.e., 0.30 syllables per second for words and 0.15 syllables per second for non-words (as reported by Tressoldi et al. [13]). IBM SPSS 25 was used for the statistical analyses and *p* was set at 0.05.

### 2.5. Ethics

All parents provided informed consent to participate and children accepted to take part in the study. All the procedures were consistent with the Declaration of Helsinki ethical principles for research involving human subjects and the study was approved by the Ethics Committee of the Policlinico San Matteo (Pavia) with number P-20200048574.

## 3. Results

### 3.1. Survey

**Figure 1:**
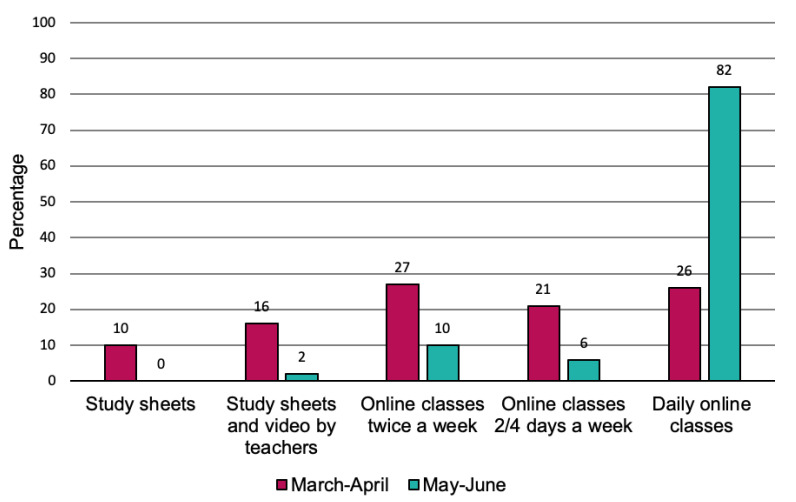
Remote school activities delivered during the first (March–April) and last (May–June) months of the lockdown period.

As showed in Figure 1, the online school activities changed during the lockdown, with an increase of live classes: during the first months of the lockdown, the majority of the students attended online live classes only twice a week and received study sheets and/or pre-recorded video by teachers as integration, while from May to June, more than 80% of them attended daily online classes.

Table 1 reports significant differences between DG and CG subjects. As expected, a higher percentage of DG children reported difficulties in following online classes and managing homework during the lockdown. Moreover, more children with dyslexia compared to CG controls perceived a worsening in various tasks, including reading, comprehension, and mathematics. These main difficulties were confirmed also by the parents’ point of view. Moreover, for DG children, greater social isolation and fewer worries about the pandemic and the school’s closure emerged.

### 3.2. Reading Skills Group Analysis

**Figure 2:**
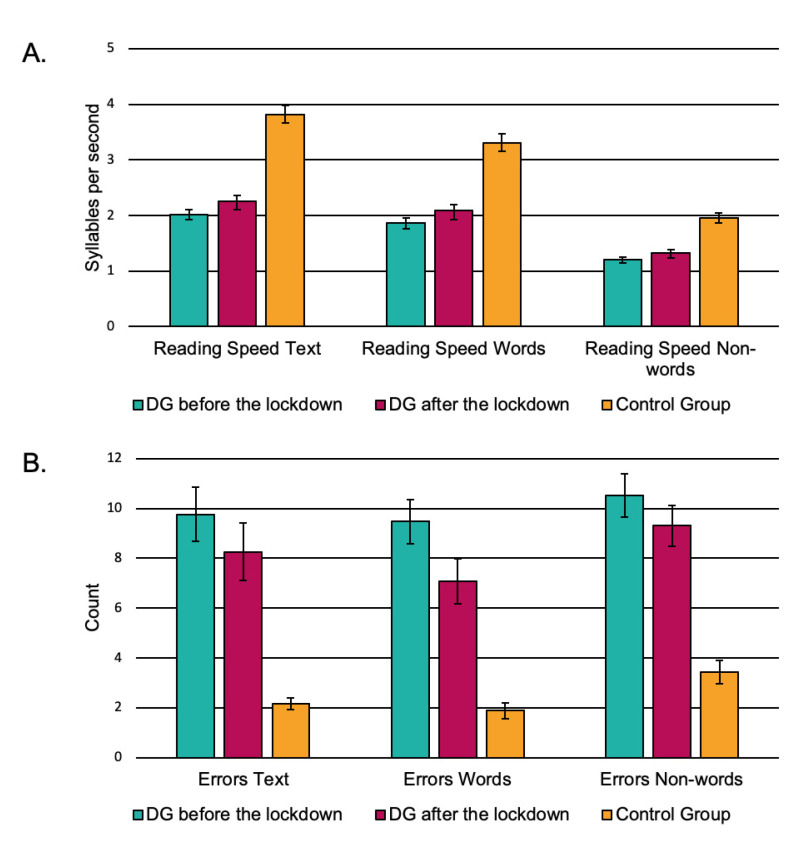
Dyslexia group (DG) and control group reading speed (**A**) and accuracy (**B**) for words, non-words, and text before and after the lockdown. Note: the control group was assessed only after the lockdown; bars represent standard errors.

DG children improved their reading speed for all the three included tasks (see Figure 2A): words t(64)= −4.99, *p* < 0.001, non-words, t(64) = −3.51, *p* = 0.001, and text, t(64) = −6.25, *p* < 0.001. DG children also showed a significant reduction of the errors’ occurrence for words, t(64) = 3.97, *p* < 0.001, non-words, t(64) = 2.25, *p* = 0.028, and text t(64) = 2.31, *p* = 0.024.

At T2, DG subjects exhibited significantly worse performance than CG counterparts for what pertains to reading speed (words: F(1,114) = 130.86, *p* < 0.001; non-words, F(1,114) = 89.32, *p* < 0.001; text: F(1,114) = 175.43, *p* < 0.001) and accuracy (words: F(1,114) = 46.16, *p* < 0.001; non-words: F(1,114) = 48.1, *p* < 0.001; text: F(1,114) = 22.82, *p* < 0.001), as showed in Figure 2.

### 3.3. Expected Improvement in Reading Speed

**Figure 3:**
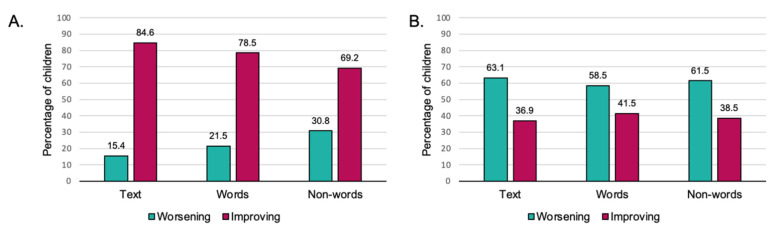
Percentages of DG children who improved after the lockdown with respect to improve-rate equal to zero (**A**) and equal to 0.30 for words and text and 0.15 for non-words (**B**). Note: DG, dyslexia group.

As showed in Figure 3A, 70% to 85% of DG children exhibited an improvement with respect to zero, suggesting that there is a naturally occurring improvement in reading speed at this age. However, a percentage from 59% to 63% did not reach the expected improvement (0.30 syllables/second for words; 0.15 syllables/second for non-words) (Figure 3B). Notably, the availability of compensatory tools and the presence of a tutor during the lockdown did not significantly affect these percentages.

## 4. Discussion

The aim of this study was to investigate the evolution of reading speed and accuracy in children with dyslexia, following the closure of schools due to the spread of the SARS-CoV-2 infection.

The first relevant finding emerging from our data is that a high percentage of children did not receive daily online live classes for a prolonged period. As expected, children with dyslexia reported more difficulties in following online classes and managing homework compared to normally developing (“healthy”) children. At the same time, however, children with dyslexia appeared to be less worried about the school closure: the school context implies the exposure to performance and social issues that usually trigger anxiety or feelings of inadequacy [14]; therefore, they may perceive the school closure as protective for them.

We also noted a failure to reach learning outcomes (in terms of expected increase of reading speed). Previous research evidenced that children with dyslexia tend to have a natural increase of reading skills, in terms both of accuracy and speed; this increase is not negligible, although significantly lower than that seen in normally developing children [13]. In keep with existing literature, we found a general significant improvement in reading speed and a reduction in the number of reading errors observed also in children with dyslexia, confirming that there is a naturally occurring improvement in reading speed at this age; however, a percentage from 59% to 63% of these children did not reach the improvement in reading skills expected in untreated children with the same diagnosis.

These data could be expected from existing literature, although the number of studies investigating the development of learning in contexts without education or with distance learning methods is low, except for those that refer to the summer break (whose duration was, however, lower than the period of school closure in Italy). Research conducted by Shynwell and Defeyter showed that during the seven-week summer vacation, children experienced a reduction of the physiological increase of learning skills, up to a total stop, but could start improving again after a similar amount of time of school frequency [15]. Alexander et al. showed that, during the summer months, the skills of children with higher socioeconomic status continued to advance (albeit, at a slower rate than the school year), whereas the skills of children with lower socioeconomic status mostly remained flat [16]. Cooper et al. suggested that skills requiring a procedural knowledge are more significantly influenced by the loss of the regular exercise provided by school frequency [17]. This could be especially relevant for children whose neuropsychological functioning towards reading is often characterized by a deficit of procedural learning [18].

The introduction of distance learning did not seem to reduce these risks. In Italy, despite regulatory prescriptions, it was realized in a way that was neither consistent nor sufficient (our findings suggest that after three months from the beginning of the lockdown, nearly one fifth of children were not receiving daily classes, despite the fact that children in our study were from Lombardy, usually considered to be one of the better organized parts of Italy). Moreover, recent data evidenced that distance learning in Italy increases educational deprivation and social inequalities, a fact more evident in children with developmental disabilities [19].

It should be also stressed that the feeling of being understood and supported by the teacher(s) is highly relevant in reducing the risk of anxiety symptoms in children with specific learning disabilities [14]. The lack of direct contact with teachers could reduce this feeling of receiving adequate attention and support, and therefore increase the risk of developing internalizing symptoms. 

Our study has some limitations. First, the control group was not assessed before the so-called “lockdown”; this prevented us from knowing the increase in reading speed obtained in these subjects, which, however, had a reading speed and accuracy in line with what expected according to their grade. Second, the control group was tested after school ending; this could, however, have only reduced the difference in reading speed and accuracy [15,16]. Third, there was a difference in terms of age between the two groups, which we were, however, able to control through statistical analyses. To end with, we did not explore the possibility of a “catch up” in terms of reading speed and accuracy after returning to a normal school frequency; this could be, however, an interesting idea for future studies.

## 5. Practical Implications

Both existing literature and our findings support the idea that children with dyslexia appear to be a specific at-risk population that may deserve tailored support during a period of therapy and school suspension. School closures carry a heavy burden of risk to reduce children’s health in a number of ways, including both biological (increased risk of overweight) and psychological (increased negative emotions and reduced discipline) aspects [20]. 

Moreover, our survey suggested that the school closure may impact also the social condition of children with learning difficulties: this could be in part a consequence of the internalizing comorbidities frequently seen in these children [21] but could also reflect an increased burden of distress caused by the feeling of an increased inadequacy coupled with a larger amount of time requested to fulfill school duties [3].

To end with, in the next years, these findings suggest increased caution in the interpretation of results of neuropsychological testing concerning reading skills, to avoid diagnostic errors which can have negative consequences in term of academic results [22] and of global well-being of the child [14].

## Figures and Tables

**Table 1:** Children and parents’ comparison about online school management. Note: DG, dyslexia group; CG, control group.

Survey Items (Children)	DG, *n* = 65	CG, *n* = 52	*χ* ^2^	*p*
Difficulties following online classes	48%	23%	6.56	<0.05
Difficulties in doing homework	62%	13%	24.4	<0.001
Perceiving that learning abilities worsened during quarantine	19%	3%	14.86	<0.01
Perceiving that reading abilities remained the same during quarantine	64%	93%	11.35	<0.05
More difficulties in text comprehension without teacher’s oral explanation	60%	33%	8.77	<0.05
Difficulties in mathematics	35%	10%	8.05	<0.01
More errors in mathematics	49%	18%	10.62	<0.01
Worsening in text comprehension	49%	18%	10.62	<0.01
More difficulties in studying	51%	13%	15.68	<0.01
Worsening in vocabulary	38%	8%	12.43	<0.01
More family conflicts	46%	20%	7.30	<0.05
Asking often when the school will reopen	19%	65%	22.36	<0.001
Missing friends	67%	93%	9.92	<0.01
Concerns about COVID-19	3%	25%	11.54	<0.01
Asking for information about COVID-19	30%	73%	32.66	<0.001
**Survey Items (Parents)**	**DG, *n* = 63**	**CG, *n* = 38**	***χ*^2^**	***p***
Difficulties following online classes	52%	0%	30.91	<0.001
Difficulties in doing homework	67%	11%	33.50	<0.001
Perceiving that reading worsened during quarantine	41%	16%	12.33	<0.05
Using more the keyboard	46%	76%	8.89	<0.01
More errors in mathematics	46%	13%	13.12	<0.05
Worsening in text comprehension	38%	5%	18.71	<0.001
Worsening in oral presentation	35%	3%	18.71	<0.001
Contac classmates for homework	11%	40%	14.81	<0.01
Teachers as emotional support	44%	82%	14.47	<0.01
Less contacts with friends	32%	11%	9.45	<0.01

## Data Availability

Raw data are available from Zenodo using DOI:10.5281/zenodo.4983550.

## References

[1] Remuzzi A., Remuzzi G. (2020). COVID-19 and Italy: What next?. Lancet.

[2] Parodi S.M., Liu V.X. (2020). From Containment to Mitigation of COVID-19 in the US. JAMA.

[3] Petretto D.R., Masala I., Masala C. (2020). School Closure and Children in the Outbreak of COVID-19. Clin. Pract. Epidemiol. Ment. Health.

[4] Bonal X., González S. (2020). The impact of lockdown on the learning gap: Family and school divisions in times of crisis. Int. Rev. Educ..

[5] American Psychiatric Association (2013). Diagnostic and Statistical Manual of Mental Disorders.

[6] Smirni P., Vetri L., Misuraca E., Cappadonna M., Operto F.F., Pastorino G.M.G., Marotta R. (2020). Misunderstandings about developmental dyslexia: A historical overview. Pediatr. Rep..

[7] Nadeau M.F., Massé L., Argumedes M., Verret C. (2020). Education for students with neurodevelopmental disabilities-Resources and educational adjustments. Handb. Clin. Neurol..

[8] Provenzi L., Grumi S., Borgatti R. (2020). Alone with the Kids: Tele-Medicine for Children with Special Healthcare Needs during COVID-19 Emergency. Front. Psychol..

[9] Asbury K., Fox L., Deniz E., Code A., Toseeb U. (2021). How is COVID-19 Affecting the Mental Health of Children with Special Educational Needs and Disabilities and Their Families?. J. Autism Dev. Disord..

[10] Grumi S., Provenzi L., Gardani A., Aramini V., Dargenio E., Naboni C., Vacchini V., Borgatti R. (2020). Engaging with Families through On-line Rehabilitation for Children during the Emergency (EnFORCE) Group. Rehabilitation services lockdown during the COVID-19 emergency: The mental health response of caregivers of children with neurodevelopmental disabilities. Disabil. Rehabil..

[11] Sartori G., Job R., Tressoldi P.E. (2007). DDE-2. Batteria per la Valutazione della Dislessia e della Disortografia Evolutiva (Battery for the Assessment of Developmental Dyslexia and Dysorthographia).

[12] Cornoldi C., Carretti B. (2016). Prove MT-3-Clinica—La Valutazione delle Abilità di Lettura e Comprensione per la Scuola Primaria e Secondaria di I Grado (MT-3—Clinic Tasks. The Assessment of Reading and Comprehension Skills for Elementary and Middle School).

[13] Tressoldi P.E., Stella G., Faggella M. (2001). The development of reading speed in Italians with dyslexia: A longitudinal study. J. Learn. Disabil..

[14] Chiappedi M., Baschenis I.M. (2016). Specific learning disorders and anxiety: A matter of school experience?. Min. Pediatr..

[15] Shinwell J., Defeyter M.A. (2017). Investigation of Summer Learning Loss in the UK-Implications for Holiday Club Provision. Front. Public Health.

[16] Alexander K.L., Entwisle D.R., Olson L.S. (2007). Summer learning and its implications: Insights from the Beginning School Study. New Dir. Youth Dev..

[17] Cooper H., Nye B., Charlton K., Lindsay J., Greathouse S. (1996). The effects of summer vacation on achievement test scores: A narrative and meta-analytic review. Rev. Educ. Res..

[18] Nicolson R.I., Fawcett A.J. (2019). Development of Dyslexia: The Delayed Neural Commitment Framework. Front. Behav. Neurosci..

[19] Chaabane S., Doraiswamy S., Chaabna K., Mamtani R., Cheema S. (2021). The Impact of COVID-19 School Closure on Child and Adolescent Health: A Rapid Systematic Review. Children.

[20] Scarpellini F., Segre G., Cartabia M., Zanetti M., Campi R., Clavenna A., Bonati M. (2021). Distance learning in Italian primary and middle school children during the COVID-19 pandemic: A national survey. BMC Public Health.

[21] Giovagnoli S., Mandolesi L., Magri S., Gualtieri L., Fabbri D., Tossani E., Benassi M. (2020). Internalizing Symptoms in Developmental Dyslexia: A Comparison Between Primary and Secondary School. Front. Psychol..

[22] Chiappedi M., Baschenis I.M., Dolci R., Bejor M. (2011). Importance of a critical reading of neuropsychological testing. Min. Pediatr..

